# Gastrocnemius Muscle Architecture in Elite Basketballers and Cyclists: A Cross-Sectional Cohort Study

**DOI:** 10.3389/fspor.2021.768846

**Published:** 2021-12-07

**Authors:** Samantha May, Simon Locke, Michael Kingsley

**Affiliations:** ^1^La Trobe Rural Health School, La Trobe University, Bendigo, VIC, Australia; ^2^Holsworth Research Initiative, La Trobe University, Bendigo, VIC, Australia; ^3^Department of Exercise Sciences, University of Auckland, Auckland, New Zealand

**Keywords:** ultrasound, fascicle length, pennation angle, muscle thickness, concentric exercise, eccentric exercise

## Abstract

Eccentric and concentric actions produce distinct mechanical stimuli and result in different adaptations in skeletal muscle architecture. Cycling predominantly involves concentric activity of the gastrocnemius muscles, while playing basketball requires both concentric and eccentric actions to support running, jumping, and landing. The aim of this study was to examine differences in the architecture of gastrocnemius medialis (GM) and gastrocnemius lateralis (GL) between elite basketballers and cyclists. A trained sonographer obtained three B-mode ultrasound images from GM and GL muscles in 44 athletes (25 basketballers and 19 cyclists; 24 ± 5 years of age). The images were digitized and average fascicle length (FL), pennation angle (θ), and muscle thickness were calculated from three images per muscle. The ratio of FL to tibial length (FL/TL) and muscle thickness to tibial length (MT/TL) was also calculated to account for the potential scaling effect of stature. In males, no significant differences were identified between the athletic groups in all parameters in the GM, but a significant difference existed in muscle thickness in the GL. In basketballers, GL was 2.5 mm thicker (95% CI: 0.7–4.3 mm, *p* = 0.011) on the left side and 2.6 mm thicker (95% CI: 0.6–5.7 mm, *p* = 0.012) on the right side; however, these differences were not significant when stature was accounted for (MT/TL). In females, significant differences existed in the GM for all parameters including FL/TL and MT/TL. Female cyclists had longer FL in both limbs (MD: 11.2 and 11.3 mm), narrower θ (MD: 2.1 and 1.8°), and thicker muscles (MD: 2.1 and 2.5 mm). For the GL, female cyclists had significantly longer FL (MD: 5.2 and 5.8 mm) and narrower θ (MD: 1.7 and 2.3°) in both limbs; no differences were observed in absolute muscle thickness or MT/TL ratio. Differences in gastrocnemius muscle architecture were observed between female cyclists and basketballers, but not between males. These findings suggest that participation in sport-specific training might influence gastrocnemius muscle architecture in elite female athletes; however, it remains unclear as to whether gastrocnemius architecture is systematically influenced by the different modes of muscle activation between these respective sports.

## Introduction

Skeletal muscle architecture is described by the arrangement of fiber bundles, known as fascicles, to the force-generating axis of pennate muscles (Wickiewicz et al., 1983; Lieber and Friden, 2000). Fascicles insert obliquely into the superficial and deep aponeuroses of a muscle which defines the pennation angle (θ), and the distance between aponeuroses defines anatomical muscle thickness. Muscle architecture can be measured *via in vivo*, two-dimensional (2D), B-mode ultrasound imaging (Kwah et al., 1985; Scholten et al., 2003; Timmins et al., 2015; Oliveira et al., 2021). Due to lower cost and greater accessibility in a variety of settings, ultrasound is popular compared to MRI, an alternative tool that requires a technique known as diffusion tension imaging (DTI) to discern individual muscle fibers (Van Donkelaar et al., 1999; Franchi et al., 2018a; Bolsterlee et al., 2019). Ultrasound measurements obtained by appropriately trained sonographers during resting or isometric conditions have been shown to be repeatable and reliable, as well as valid against historic measures based on microdissection of whole cadaveric muscles (Kwah et al., 1985; Reeves and Narici, 1985; Ando et al., 2014). Once images are acquired by a trained sonographer, measurements can be made using manual digitization with custom-written computer software (Narici et al., 1996; Maganaris et al., 1998; Aagaard et al., 2001; Chleboun et al., 2001, 2007; Muramatsu et al., 2001; Boer et al., 2008; Aggeloussis et al., 2009; Raj et al., 2012a; Gillett et al., 2013; Lidstone et al., 2016), or automated tracking (Cronin et al., 1985; Rana et al., 2009; Zhou et al., 2014; Drazan et al., 2019).

Skeletal muscle architecture parameters have been measured in studies of muscle physiology and biomechanics. The geometric arrangement determines muscle length and the length range over which active force can be generated, which influences contraction velocity, force-generating capacity, and the functional excursion of the muscle (Wickiewicz et al., 1983; Blazevich et al., 1985; Narici et al., 1996; Fukunaga et al., 1997; Kawakami et al., 1998; Maganaris et al., 1998; Lieber and Friden, 2000, 2001; Hodges et al., 2003; Blazevich, 2006; Kruse et al., 2021). Muscle length is determined by fascicle length (FL) which is defined by the number of sarcomeres in-series, as well as by θ (Kruse et al., 2021). Recent studies have found that certain architectural characteristics relate to advantages in physical performance. Longer FL has been associated with sprint performance, as the number of sarcomeres arranged in-series is proportional to the maximum shortening velocity of the muscle (Abe et al., 2000; Kumagai et al., 2000; Blazevich, 2006). Greater θ allows a greater amount of contractile tissue to attach to a given area of tendon or aponeurosis within a cross-sectional area and predicts the maximal capacity for force production (Kawakami et al., 1993; Fukunaga et al., 1997; Bamman et al., 2000; Lieber and Friden, 2001; Blazevich and Sharp, 2005). Muscle thickness correlates to muscle cross-sectional area which is proportional to the number of sarcomeres in-parallel, influencing maximal force production (Lieber and Friden, 2000; Narici et al., 2016). Consequently, architectural adaptations following specific modes of training have become an area of interest.

Isotonic skeletal muscle actions are defined by changing muscle length whilst tension remains unchanged (Hill, 1925). There are two basic types of isotonic actions (concentric and eccentric). During concentric action, muscle tension is increased to meet resistance then remains stable as the muscle shortens, generating force *via* the tendon complex which results in joint movement (Padulo et al., 2013). Eccentric muscle action occurs when a force applied to the muscle exceeds the force produced by the muscle itself, resulting in a lengthening action of the muscle-tendon complex and absorption of mechanical energy which is either lost as heat or stored as elastic potential energy for subsequent concentric actions (Lindstedt et al., 2001; LaStayo et al., 2003). Overall, eccentric actions can produce greater muscle force (Hortobagyi and Katch, 1990) with a lower metabolic energy and oxygen cost (Abbott et al., 1952; LaStayo et al., 2003). Training interventions that selectively assigned participants to eccentric or concentric actions resulted in different morphological adaptations in muscle architecture (Franchi et al., 2014, 2015, 2017; Hoppeler, 2016; Kruse et al., 2021). Authors of a recent narrative review concluded that concentric training might increase muscle length due to the addition of sarcomeres in-parallel within the muscle fibers, whereas eccentric training may result in longer fascicles and thus longitudinal muscle growth due to the addition of sarcomeres in-series (Kruse et al., 2021). The intensity of training and the length range at which the muscle is active is also thought to influence muscle architecture adaptations, however, the mechanism and degree to which this occurs are not clear (Kruse et al., 2021). Interpretation of findings from past studies should follow the principle of specificity, meaning that the training responses described are tightly coupled to the muscle group and the muscle fibers that have been recruited during the training.

Training adaptations to gastrocnemius muscle architecture have been explored previously due to its contribution to the functional demands of locomotion as part of the triceps surae muscle group, as well as the convenience and ease of examination under ultrasound. The gastrocnemius medialis (GM) and gastrocnemius lateralis (GL), along with the soleus, are the greatest contributors to propulsion for walking and running in mid to late stance (Sasaki and Neptune, 2005; Hamner et al., 2010), ankle power release required in jumping (Bobbert et al., 1986; Kurokawa et al., 2001), and the support of body weight during landing (Farris et al., 2016). Highly trained athletes who repeat specific movement patterns might have specific muscle architecture profiles related to the physical requirement of their training (Savelberg and Meijer, 1985; Brughelli et al., 2010). Alongside the vastus medialis and vastus lateralis (VL), the GM and GL are the most activated muscles during cycling on a bicycle (Ericson et al., 1985). Athletes specialized in conventional cycling, where the gastrocnemius is predominantly activated concentrically (Ericson et al., 1985; Bijker et al., 2002), could have structural adaptations different to locomotive sports athletes. Basketball is a locomotive sport where the gastrocnemius muscle of an athlete is activated eccentrically as well as concentrically, at different joint angles and muscle lengths during running, sprinting, accelerating, sudden stopping, changing direction, jumping, and landing (Savelberg and Meijer, 1985; Vogt and Hoppeler, 1985; Ben Abdelkrim et al., 2007; Ullrich and Brueggemann, 2008). Throughout locomotion, eccentric actions of the gastrocnemius muscles support the weight of the body against gravity particularly during body deceleration, by generating force while lengthening and exerting a breaking action against downward movement (LaStayo et al., 2003; Gault and Willems, 2013; Isner-Horobeti et al., 2013). The lengthened muscle-tendon system converts absorbed mechanical energy into stored elastic recoil energy for subsequent concentric actions of the gastrocnemius during limb propulsion, which results in less muscle work and energy required in running or plyometric activities such as jumping, landing, or changing direction (Vogt and Hoppeler, 1985; LaStayo et al., 2003; Konow and Roberts, 2015; Farris et al., 2016). During running, the gastrocnemius muscles experience higher loads during a forefoot strike pattern, to attenuate the impact energy associated with eccentrically controlling the ankle dorsiflexion moment stimulus (Gruber et al., 2014; Yong et al., 2020). This eccentric phase to dissipate impact and reduce shock is absent in cycling.

Past studies have examined the effects of dedicated eccentric or concentric training on muscle architecture, but there is a paucity of studies specific to the gastrocnemius muscle which also differ in results. Two studies found that eccentric training increased FL and muscle thickness but had conflicting θ findings (Duclay et al., 2009; Geremia et al., 2019). Another found similar results to previous studies that examined the VL (Franchi et al., 2015, 2018b), reporting that eccentric or concentric training produced similar increases in muscle thickness (English et al., 2014). The comparable increases in muscle thickness between the two types of training could be due to distinct structural adaptations (Franchi et al., 2017), such as greater FL increases following eccentric training, vs. greater θ increases after concentric training (Franchi et al., 2014, 2015, 2018b). However, this adaptation has not been consistently reported in studies of GM. Other studies found no change in GM architecture at all following both types of training (Foure et al., 1985; Raj et al., 2012b). It cannot be assumed that the changes seen in the GM will be consistent with the GL. Whilst they are synergists, these muscles have differences in muscle-tendon anatomy and joint articulation. They receive different activation messages and have different force-generating capacities due to their different architectural properties (Crouzier et al., 2018; Lai et al., 2018). GL is characterized by longer FLs and smaller θs whereas the GM is characterized by shorter FLs and larger θs (Koryak, 1985; Kawakami et al., 1998). Two studies found that shorter FLs are more susceptible to muscle damage caused by eccentric training (Proske and Morgan, 2001; Baroni et al., 2013). Consequently, each muscle should be examined separately due to its specific adaptive responses.

The aim of this study was to explore the differences in muscle architecture of the GM and GL muscles at rest, between two cohorts of athletes; one group of endurance cyclists that train with primarily concentric muscle actions compared to one group of basketballers that train with a combination of eccentric and concentric muscle actions. To account for the effects of mechanical differences that might result from the unilateral forces that apply to jumping athletes who repetitively and forcibly jump off one leg, we assessed muscle architecture in both left and right limbs to ensure that any group differences were not due to habitual unilateral loading associated with limb dominance (Bohm et al., 2015; Bayliss et al., 2016). The authors hypothesized that differences in muscle architecture arrangements between the two groups of athletes would exist for both genders.

## Materials and Methods

### Study Design and Participants

The study group consisted of 44 adult athletes who volunteered to participate in this study and signed written informed consent (Table 1).

**Table 1:** Participant characteristics (mean ± SD).

		**Females**			**Males**		
		**BB**	**CYC**	**Total**	**BB**	**CYC**	**Total**
	**Number**	**13**	**10**	**23**	**12**	**9**	**21**
Age	(year)	27 ± 6	24 ± 4	26 ± 6	25 ± 5	24 ± 4	25 ± 5
Body mass	(kg)	76.8 ± 17.7	64.4 ± 9.3	71.4 ± 15.6	91.6 ± 13.7	76.0 ± 9.7	84.9 ± 14.3
Stature	(cm)	174.9 ± 8.1	166.5 ± 5.8	171.3 ± 8.2	192.7 ± 6.5	179.3 ± 5.4	187.0 ± 9.0
Tibial Length	(cm)	39.2 ± 2.6	37.7 ± 2.2	38.1 ± 2.8	44.2 ± 5.0	41.0 ± 2.1	42.8 ± 4.2
Training experience	(years)	11.4 ± 4.4	5.6 ± 5.7	8.9 ± 5.7	8.3 ± 5.4	8.8 ± 5.9	8.5 ± 5.5
Compete ≥ state level	(%)	100%	50%	78%	100%	55%	81%

*BB, basketballer; CYC, cyclist*.

Participants were screened for eligibility using a questionnaire that covered demographic information such as age, dominant leg, training experience, and competition experience measured in years, current training amount per week, level of competition, and previous history of lower limb injury. Athletes had on average 8.7 ± 5.5 years of experience in their sport and trained at a minimum of at least three times per week, including competition. Participants were excluded if they did not perform their sport at least three times per week; had any history of an acute or chronic lower limb injury within the previous 12 months; or if they had exercised using their calf muscles during the same day prior to the assessment, including jogging, running, sprinting, hopping, skipping, jumping, or performing heel raises. The study was approved by La Trobe University Human Ethics Committee (Application Number: S17–114) and was conducted according to the National Statement on Ethical Conduct in Human Research.

### Procedures

Body mass was measured to the nearest 0.1 kg using a calibrated analog floor-scale (Model 762; Seca, Germany); stretch stature was measured to the nearest 0.1 cm using a portable stadiometer (Model 213; Seca; Germany) according to the procedures described by the International Society for the Advancement of Kinanthropometry (Marfell-Jones et al., 2012). The tibial length was estimated using validated regression equations based on stature (Saco-Ledo et al., 2019). Skeletal muscle architecture of the GL and GM muscles at rest was measured using 2-dimensional B-mode ultrasound (LOGIQ V2; GE Healthcare, Australia), with a 38 mm wide linear probe and a standardized frequency of 12–13 MHz.

**Figure 1:**
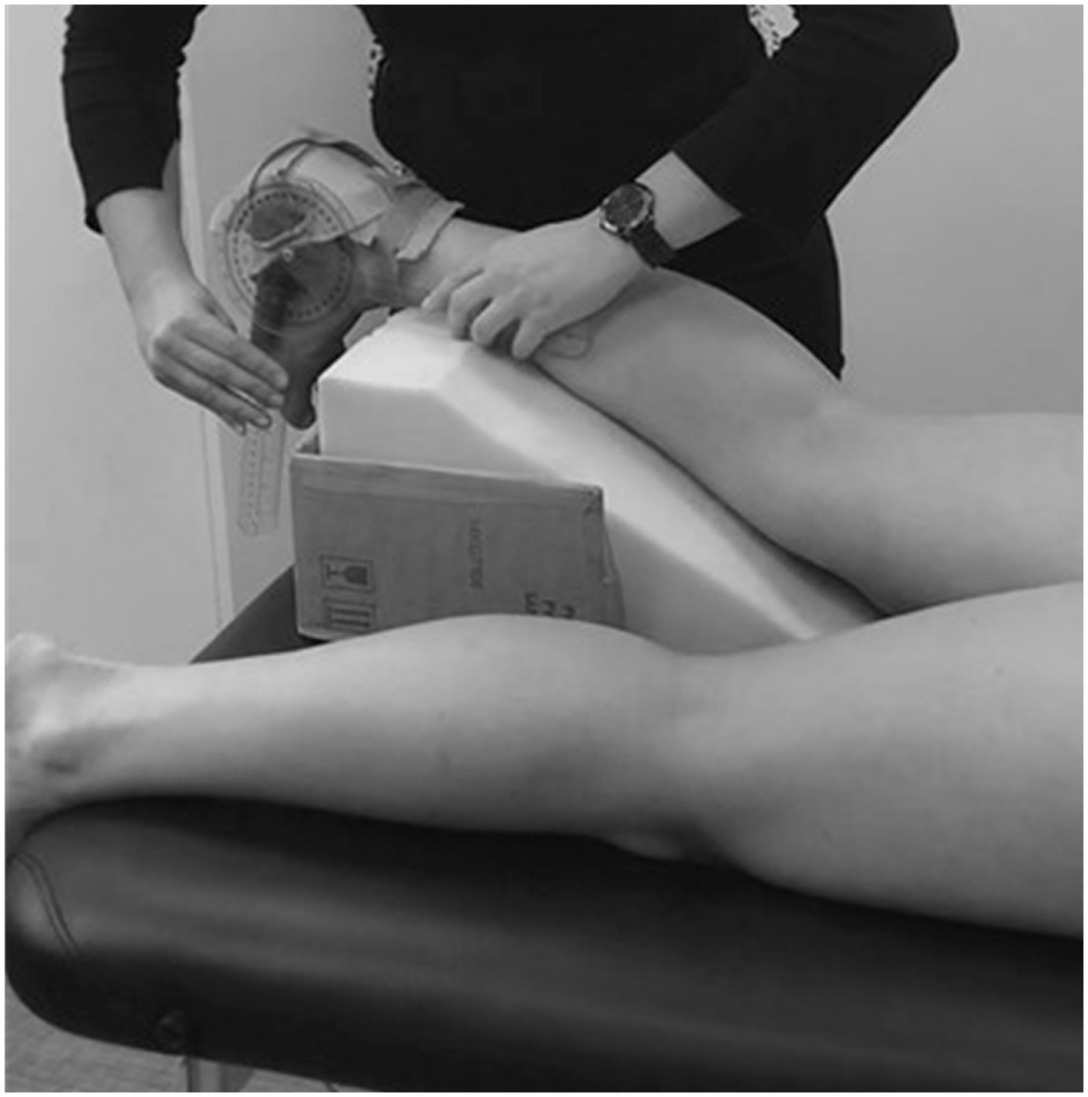
Position of the lower limb during ultrasonography.

**Figure 2:**
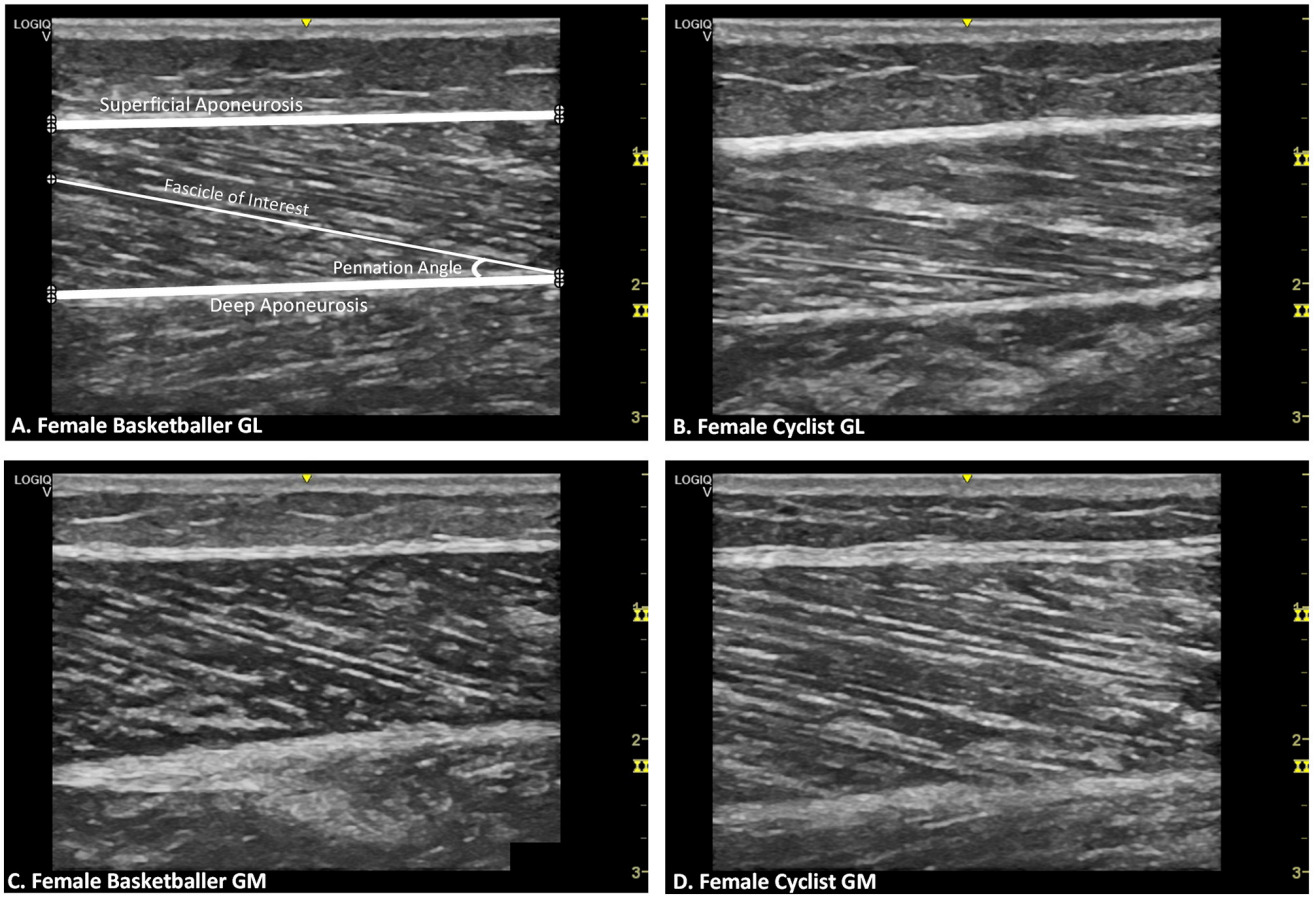
Representative B-mode ultrasound images for female athletes by muscle and training group. **(A)** Gastrocnemius lateralis (GL) of female basketballer Points of interest plotted on the image (⊕) are for superficial aponeurosis borders, deep aponeurosis borders, and a selected fascicle length with the corresponding pennation angle. **(B)** GL of female cyclist. **(C)** Gastrocnemius medialis (GM) of female basketballer. **(D)** GM of female cyclist.

Each participant lay prone on an examination table and an incline foam wedge was used to support their flexed knee up to 30° to ensure the gastrocnemius muscle was relaxed at the knee joint. A custom splint secured the ankle joint close to 90° where the sole of the foot is perpendicular to the tibia (Figure 1). This position was confirmed with a manual goniometer and a position within the range of 85° to 95° was accepted. The sonographer identified the probe site at one-third of the distance from the popliteal crease of the knee to the tip of the medial malleolus for the GM, and the lateral malleolus for the GL (Koryak, 1985; Kawakami et al., 1998; Kurokawa et al., 2001; Legerlotz et al., 2010; Raj et al., 2012a; Cho et al., 2014; Konig et al., 2014). The transducer probe was positioned perpendicular to the long axis of the leg, at the midpoint of the GL and GM muscle bellies, found at the center of each muscle halfway between its medial and lateral borders (Koryak, 1985; Kawakami et al., 1998; Kurokawa et al., 2001; Legerlotz et al., 2010; Raj et al., 2012a; Cho et al., 2014; Konig et al., 2014). These sites were chosen as previous studies concluded that this level is where the anatomical cross-sectional area of the muscle is maximal (Fukunaga et al., 1992; Kawakami et al., 1998); it best aligns the image plane with muscle fascicles (Benard et al., 2009; Bolsterlee et al., 2016); there is minimal fascicle curvature at these sites with participants at rest (Raj et al., 2012a). While holding the transducer at a single site on the skin, manipulation, and rotation of the probe around the sagittal-transverse axis was performed by the investigator to ensure the superficial and deep aponeuroses were as parallel as possible and to optimize the visibility of the fascicles as continuous striations from one aponeurosis to the other (Bolsterlee et al., 2016). Care was taken to ensure that minimal compression by the ultrasound probe on the skin occurred, and transmission gel was applied to improve acoustic coupling. Three ultrasound images were captured from each GM and GL at the left and right limb within one session, totaling 12 images per participant. These images were recorded digitally and sent to an external investigator for de-identification and randomization (www.randomizer.org). The de-identified and randomized images were analyzed using novel computer software designed in LabVIEW (version 16; National Instruments, USA). One investigator performed the digitization of the blinded images, and from each image, three fascicles, three θs, and a single measure of muscle thickness were calculated. Figure 2, panel A shows the points of interest plotted during manual digitization. As three images were taken per muscle, this gave a total of nine FLs, nine θs, and three measures of muscle thickness for each muscle belly. From there, the average was calculated, and this final measure was used in the statistical analysis. Furthermore, the ratio between FL (cm) to tibial length (m) (FL/TL), and the ratio between muscle thickness (cm) to tibial length (m) (MT/TL), were used to explore any effects of scaling on stature differences between the two comparison groups and normalize architectural measurements to limb length (Kubo et al., 2003).

Intra-rater test-retest reliability analysis for this method of digitization using a single investigator was performed prior to the study with 100 images digitized on two separate occasions and showed excellent test-retest reliability with an ICC of 0.99 (95% CI: 0.98, 0.99, *p* < 0.001) for FL, and 0.99 (95% CI: 0.98, 0.99, *p* < 0.001) for θ (May et al., 2021).

### Statistical Analysis

All data were assessed for normality using the Shapiro-Wilk test, and data were normally distributed.

To explore our first hypothesis, a mixed-model ANOVA (within factors: athletic group and limb; between factor: gender) was conducted to examine the effect of gender (male vs. female), group (basketballer vs. cyclist), and limb (left vs. right) on absolute muscle architecture values and relative to tibial length values. Where there was a significant three-way or two-way interaction effect, simple main effects were evaluated by limb, gender, and group. Pearson Product Moment correlation was performed to explore relationships between age, body mass, stature, and training years with FL, θ, and muscle thickness, respectively. Statistical analyses were performed using IBM SPSS version 25 (Armonk, NY: IBM Corp., USA). Group data are presented as mean ± standard deviation (*SD*) and statistical significance was set at *p* ≤ 0.05.

## Results

Table 2 displays muscle architecture data for basketballers and cyclists by gender and limb. Figure 2 provides representative ultrasound images of female GM and GL muscles for both athlete groups.

**Table 2:** Summary of gastrocnemius architectural data in basketballers and cyclists (mean ± SD).

		**Gastrocnemius lateralis**			**Gastrocnemius medialis**		
		**Basketballers**	**Cyclists**	**Difference MD (95% CI)**	**Basketballers**	**Cyclists**	**Difference MD (95% CI)**
Fascicle length (mm)	Male, Left	74.2 ± 7.2	65.3 ± 16.0	8.9 (−6.5–24.3)	59.6 ± 12.7	52.8 ± 7.4	6.8 (−2.5–16.1)
	Male, Right	82.1 ± 14.6	75.2 ± 16.8	6.9 (−7.4–21.3)	60.5 ± 13.6	53.2 ± 8.7	7.3 (−3.6–18.2)
	Female, Left	66.1 ± 13.0	82.5 ± 17.8	16.3 (3.0–29.6)*	52.6 ± 6.6	63.8 ± 10.5	11.2 (3.5–18.9)*
	Female, Right	64.6 ± 10.9	80.9 ± 14.2	16.3 (5.4–27.2)*	51.3 ± 1.6	62.6 ± 8.7	11.3 (4.9–17.6)*
FL/TL (ratio: cm/m)	Male, Left	16.8 ± 3.2	16.4 ± 4.2	0.8 (−2.6–4.1)	13.5 ± 2.5	12.9 ± 1.8	0.6 (−1.5–2.7)
	Male, Right	18.7 ± 3.1	18.4 ± 4.2	0.3 (−3.1–3.6)	13.7 ± 2.3	13.0 ± 2.3	0.6 (−1.5–2.8)
	Female, Left	16.8 ± 2.9	22.0 ± 5.2	5.2 (1.2–9.1)*	13.6 ± 1.6	17.6 ± 3.5	4.0 (1.4–6.6)*
	Female, Right	16.5 ± 2.6	22.3 ± 4.9	5.8 (2.1–9.5)*	13.2 ± 1.3	17.2 ± 3.1	4.0 (1.7–6.2)*
Pennation Angle (°)	Male, Left	14.5 ± 2.7	12.7 ± 3.4	1.9 (−0.9–4.7)	23.6 ± 3.8	23.2 ± 3.5	0.5 (−2.9–3.9)
	Male, Right	12.5 ± 2.3	11.8 ± 2.7	0.7 (−1.6–3.0)	21.0 ± 3.7	22.8 ± 4.0	1.8 (−1.7–5.4)
	Female, Left	11.7 ± 1.7	10.0 ± 1.6	1.7 (0.7–2.6)*	19.5 ± 0.8	17.4 ± 1.8	2.1 (0.1–4.1)*
	Female, Right	12.4 ± 0.5	10.1 ± 1.5	2.3 (0.9–3.7)*	19.5 ± 1.5	17.7 ± 1.3	1.8 (0.6–3.1)*
Muscle thickness (mm)	Male, Left	15.6 ± 2.6	13.1 ± 1.3	2.5 (0.7–4.3)*	21.0 ± 3.0	19.0 ± 2.7	2.1 (−0.6–4.7)
	Male, Right	16.2 ± 2.4	13.6 ± 1.7	2.6 (0.6–5.7)*	20.1 ± 2.5	19.5 ± 2.9	0.6 (−1.8–3.1)
	Female, Left	11.9 ± 1.8	12.4 ± 1.7	1.0 (−0.6–2.5)	15.9 ± 2.2	18.0 ± 2.8	2.1 (0.1–4.2)*
	Female, Right	12.6 ± 2.2	12.7 ± 2.2	0.0 (−1.9–1.9)	16.0 ± 2.0	18.5 ± 3.2	2.5 (0.2–4.8)*
MT/TL (ratio: cm/m)	Male, Left	3.6 ± 0.6	3.2 ± 0.4	0.3 (−0.2–0.8)	4.8 ± 0.7	4.7 ± 0.7	0.1 (−0.5–0.8)
	Male, Right	3.7 ± 0.4	3.3 ± 0.5	0.3 (−0.1–0.8)	4.6 ± 0.4	4.8 ± 0.9	0.2 (−0.5–0.9)
	Female, Left	2.9 ± 0.3	3.4 ± 0.6	0.5 (0.1–0.9)	4.1 ± 0.5	4.9 ± 0.9	0.8 (0.2–1.5)*
	Female, Right	3.2 ± 0.6	3.5 ± 0.8	0.3 (−0.3–0.8)	4.1 ± 0.5	5.1 ± 1.0	0.9 (0.2–1.7)*

FT/TL, ratio of fascicle length (mm) and tibial length (mm) × 100; MT/TL, ratio of muscle thickness (mm) and tibial length (mm) × 100; MD (95% CI), mean difference between athletes with 95% confidence intervals; *p < 0.05, significantly different.

### Fascicle Length

**Figure 3:**
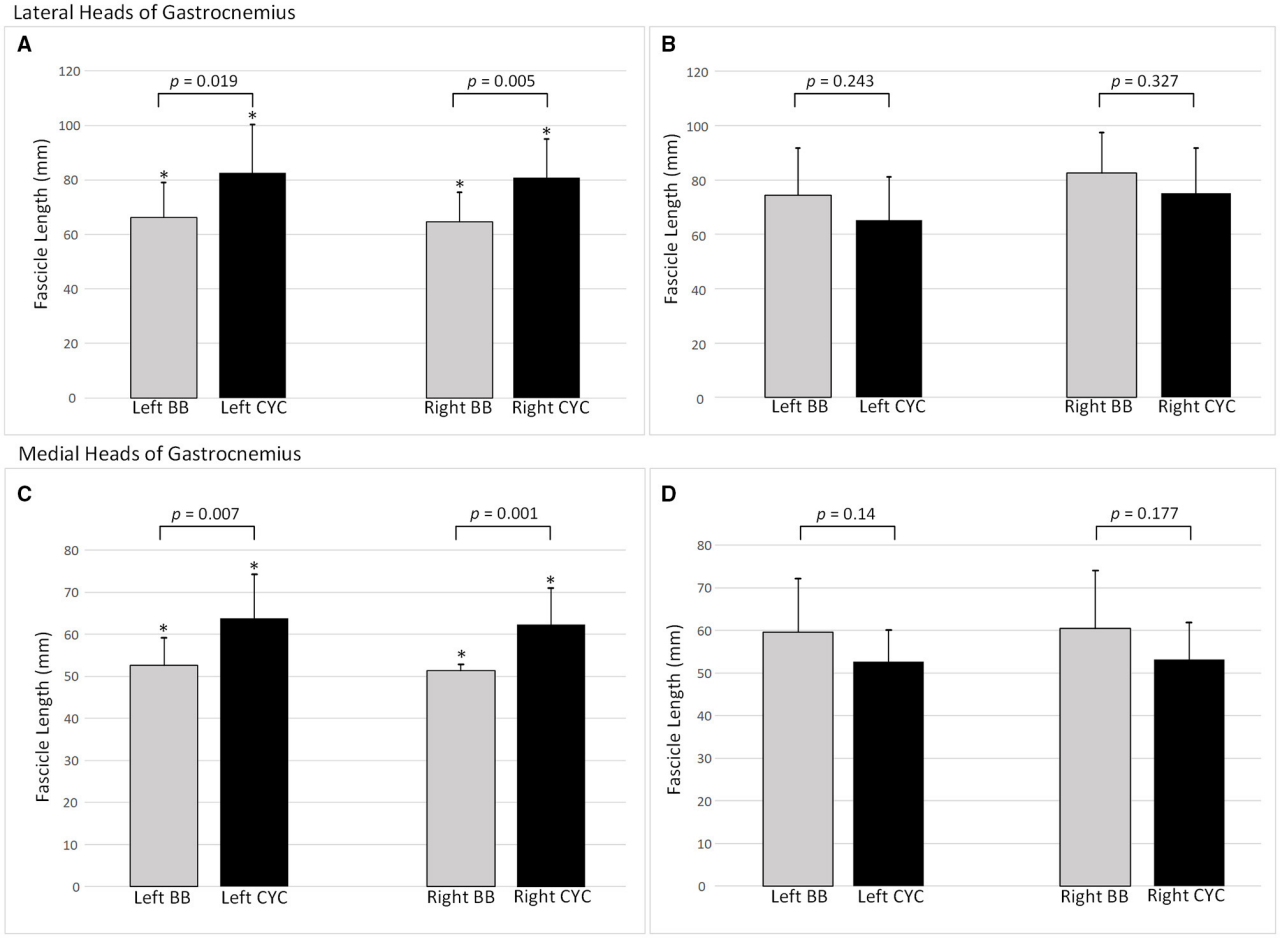
Fascicle lengths by training group and limb side. Mean ± *SD*. **(A)** Female lateral heads of gastrocnemius fascicle lengths (FLs); **(B)** Male lateral heads of gastrocnemius FLs; **(C)** Female medial heads of gastrocnemius FLs; **(D)** Male medial heads of gastrocnemius FLs. BB, basketballer; CYC, cyclist; *p-value indicates statistical significance.

For GL, significant two-way interaction effects existed for both absolute FL and FL/TL (*F*_(1, 40)_ ≥ 7.586, *p* ≤ 0.009). In the female group, cyclists had longer absolute FL compared to basketballers with a difference of 22% for both left and right limbs (*p* ≤ 0.019; Figure 3). Relative to tibial length, the intergroup differences for the left and right limbs were 27 and 30%, respectively (*p* ≤ 0.015; Table 2).

Two-way interactions were significant for both absolute FL and FT/TL in GM (*F*_(1, 39)_ ≥ 9.971, *p* ≤ 0.003). Female cyclists had 19% longer FL in the left limb (*p* = 0.001), and 20% longer FL in the right limb (*p* = 0.007; Figure 3). This pattern remained for FL/TL ratio comparisons, with the difference increasing to 26% on both sides (*p* ≤ 0.006; Table 2).

There were no statistically significant differences in FL or FL/TL between groups in male athletes for either GM or GL (Table 2).

### Pennation Angle

For GM, female basketballers had left limb θ mean ± SD values of 19.5° ± 1.5°, and right limb θs of 19.5° ± 0.8°, which were 11% and 10% greater than the cyclists who had left limb θs of 17.4° ± 1.8°, and right θs of 17.7° ± 1.3° (*p* ≤ 0.05; Table 2). For GL, basketballers had greater θs by 16% on the left and 20% on the right (*p* ≤ 0.003; Table 2).

There were no significant differences in θ values for both the GL and GM muscles between athletic groups in the males (Table 2).

### Muscle Thickness

For GL and GM, significant two-way interaction effects existed (*F*_(1, 39)_ ≥ 4.298, *p* ≤ 0.045).

Male basketballers had a greater absolute muscle thickness in GL than cyclists by 17% for the left limb (*p* = 0.011) and 20% for the right (*p* = 0.012; Table 2), but no significant differences were seen for the GM. The ratio of MT/TL for GL and GM was not significantly greater in male basketballers compared to cyclists. The GM absolute muscle thickness and MT/TL ratio were significantly greater in female cyclists compared to female basketballers (*p* ≤ 0.037; Table 2). No significant differences were observed in absolute muscle thickness and MT/TL ratio in the GL of female athletes.

### Correlations

For all athletes, there were no significant bivariate correlations between age or training time in years, with FL, θ, or muscle thickness for the GL or the GM.

For the GL, moderate correlations existed between body mass and FL in both right and left limbs for males (*r* ≥0.531, *p* ≤ 0.013), and between body mass and muscle thickness (*r* ≥0.435, *p* ≤ 0.048), but not for θ. Moderate correlations existed between stature and muscle thickness for males (*r* ≥0.529, *p* ≤ 0.014), but not for FL or θ. No significant correlations existed for GL in the female athletes.

For the GM, moderate correlations existed between body mass and FL in both right and left limbs for males (*r* ≥ 0.471, *p* = 0.031) but not for female athletes. A moderate correlation existed between body mass and muscle thickness in the right and left limbs of males (*r* ≥0.468, *p* ≤ 0.032). No significant correlations existed between body mass and θ in males and females. Between stature and FL, a moderate correlation existed for males (*r* ≥0.419, *p* ≤ 0.059) but not females, and there was no significant correlation between stature and θ, or between stature and muscle thickness.

## Discussion

In comparison with female basketball players, female cyclists had longer FL and smaller θ at the mid-belly in GM and GL as well as greater muscle thickness in the GM. These differences remained significant when FL and muscle thickness were normalized relative to tibial length. In males, differences in FL, θ, and muscle thickness of GM between cyclists and basketballers were not evident, and differences in GL muscle thickness were not significant when normalized relative to tibial length. Although differences can exist in the muscle architecture of GM and GL between athletic populations, the disparity in findings between genders suggests that long-term exposure to sports-specific training does not systematically influence GM or GL muscle architecture.

To our knowledge, this is the first study to compare the architecture of GM and GL in trained female and male cyclists and basketballers. The GM FL in female basketballers was similar in length to findings of a previous cross-sectional study that examined the GM architecture of female volleyball players. With an average FL of 47.4 mm (Donti et al., 2019), the volleyball players had FLs that were in similar lengths to the basketballers and shorter than the cyclists in our study. Volleyball is a sport that shares some common physical requirements as basketball as it includes a mixture of eccentric and concentric training methods, which include explosive jumping, landing, acceleration, agility, and periodic resistance training. Another cross-sectional study identified that female sprinters had FL of 74.4 ± 10.7 mm for the GL and 59.2 ± 7.7 mm for the GM, which were significantly longer than the study control group (62.6 ± 8.7 and 55.2 ± 6 mm for GL and GM, respectively) (Abe et al., 2001). Longer fascicles are thought to improve sprint performance due to increased contraction shortening velocity (Abe et al., 2000; Kumagai et al., 2000). However, the female cyclists in this study, who engaged in endurance training with the concentric activity of their gastrocnemius, had FLs that were consistently longer than the female basketballers, and these differences in FL were maintained when FL was presented as a ratio of tibial length (FL/TL). Interestingly, the female cyclists in the current study had similar anthropometric characteristics (stature: 163 ± 6 cm; tibial length: 37.9 ± 2.6 cm; body mass: 54.9 ± 4.9 kg) as the female sprinters in the aforementioned study (Abe et al., 2001).

In males, no significant differences were identified in FL or FL/TL of GM and GL between cyclists and basketballers. Lee et al. (Lee et al., 2021) explored architectural differences between sprint cyclists and endurance cyclists and found no significant differences in FL for both GM and GL. For GL, the mean FLs of the male athletes in this study were more closely matched to male sprinters from previous studies (GL: 80.4 ± 14.7 mm) than male endurance cyclists (GL: 54.7 ± 11.8 mm), male sprint cyclists (GL: 54.5 ± 19.1 mm) and male endurance runners (GL: 62.3 ± 10.7 mm) of a similar age (Abe et al., 2000; Lee et al., 2021). Although it is possible that the intermittent nature of basketball might explain the similarity in FL between basketballers and sprinters, FL in the male endurance cyclists was not different from the basketballers in this study. Furthermore, the mean FL of the GM of the male athletes was more closely matched to male endurance runners (GM: 53.6 ± 7.2 mm) (Abe et al., 2000), endurance cyclists (GM: 56.2 ± 7.7 mm), and sprint cyclists (GM: 57.2 ± 11.5 mm) (Lee et al., 2021) compared to male sprinters (GM: 66.4 ± 13.2 mm) (Abe et al., 2000). The lack of consistency in the differences between cycling and basketball athletes in FL and FL/TL across genders suggests that different muscle activation modes in select sports are unlikely to systematically influence FL.

This study found that female cyclists had thicker muscles in the GM, and there were no significant differences in the GM thickness of male athletes. Past studies have reported significant links between muscle thickness and muscle strength (Abe et al., 2001), with longitudinal studies supporting increased muscle thickness correlating to greater power output as seen in the anterior thigh and gastrocnemius muscles of sprinters (Abe et al., 2000). Specific to the gastrocnemius, Lee et al. (Lee et al., 2021) reported that GM muscle thickness was significantly greater in male sprint cyclists (GM: 20.9 ± 4.1 mm) compared to endurance cyclists (GM: 15.9 ± 3.2 mm) and that this was correlated to cycling power over 20 s. The study of Abe et al. (Abe et al., 2000) found GM thickness of male sprinters (GM: 23.9 ± 3.4 mm), was significantly greater than endurance runners (GM: 21 ± 2.4 mm). In basketball games where physical demands are intermittent and high intensity, ~10% of movements are short sprints up to 20 m (McInnes et al., 1995). The male sprinters from the previous study did have thicker gastrocnemius muscles than the male basketball players in this study, and male sprint cyclists from the previous study did have thicker gastrocnemius muscles compared to the male endurance cyclists analyzed by this study, however, we did not determine a difference in GM adaptive responses to training between male basketballers and endurance cyclists. The inconsistency in adaptive responses for the GM between genders confirms that muscle hypertrophy can be equally associated with both concentric only and concentric/eccentric training, a finding supported by previous studies (Seger et al., 1998; English et al., 2014; Franchi et al., 2014, 2015, 2017). For the GL, no significant differences in GL thickness between female basketballers and cyclists existed. For males, although basketballers had significantly thicker GL muscles, the difference in the GL normalized once standardized against tibial length, which suggests that muscle thickness can be proportional to stature. Moderate-strength correlations have been reported between stature and body mass with both FL and muscle thickness. Accounting for stature differences with scaling or attempts to match groups for stature and body mass should be performed in future studies (Kubo et al., 2003; Morse et al., 2005). Unlike the GM, the study of Lee et al. (Lee et al., 2021) found no significant differences in GL thickness between male sprint cyclists (GL: 13 ± 2.6 mm) and endurance cyclists (GL: 13.7 ± 5.2 mm). The work of Geremia et al. (Geremia et al., 2019) also found that the GL muscle showed the smallest adaptability to eccentric training compared to GM and soleus, the calf synergists. This confirms that adaptive responses to the GM and the GL are likely to be muscle-specific. Greater micro-damage to GM could occur following either type of concentric or eccentric/concentric training. The GM and GL should be separately studied when determining the effects of training (Koryak, 1985; Kawakami et al., 1998).

Regarding θ, the pattern of results in our male population supports previous findings that hypertrophied muscles have greater θ (Kawakami et al., 1993) as seen particularly in sprinters. However, the differences in θ between male basketballers and cyclists were not significant for either the GM or GL. Interestingly, the male θ for the GM was closer to sprint (20.3 ± 3.7°) than endurance (17.2 ± 2.8°) cyclists from a previous study (Abe et al., 2000), and like our study, found no difference in GL angle between the two groups. Paradoxically, this study found that female cyclists had smaller θ with thicker muscles, and this was significantly different to female basketballers. Compared to female sprinters, female basketball players had narrower muscles compared to sprinters (21.2 mm in GM and 16.9 mm in GL) but had similar θ to sprinters (21.1° in the GM and 13.1° in the GL) (Abe et al., 2001). Overall, this study found that θ does not appear to be systematically influenced by sports-specific training, nor systematically related to muscle thickness. Past longitudinal study reports of θ adaptations following short-term periods of resistance training in various muscle groups have also been conflicting. Some studies have shown greater increases in θ with concentric-only (Franchi et al., 2014, 2015, 2018b), or conventional concentric and eccentric resistance training (Reeves et al., 2009), others with eccentric-only training (Duclay et al., 2009), and some finding equally increased θs following both types of muscle actions (Blazevich et al., 1985) or no change at all (Raj et al., 2012b; Baroni et al., 2013). This may be due to the lower reliability of θ measurements using ultrasound compared to FL or muscle thickness (Klimstra et al., 2006; Baroni et al., 2013; Geremia et al., 2019).

Ultrasound is an operator-dependent tool, and potential sources of measurement error stem from probe placement or location, probe pressure, and probe orientation (Klimstra et al., 2006; Konig et al., 2014), which can lead to over- or underestimation of θ (Benard et al., 2009; Konig et al., 2014). The best practice is to place the probe parallel to the plane of the fascicle, however past studies have found that small manipulations of the orientation and rotation of the ultrasound probe can result in a 12% difference (13.6–15.5°) in the θ reported, and this measurement error is close to the reported adaptive response of θ to resistance training (Klimstra et al., 2006; Baroni et al., 2013; Geremia et al., 2019). To overcome limitations with ultrasonography use, this study used a single skilled sonographer who took care with applied pressure during probe placement to avoid potential tissue deformation (Konig et al., 2014). Probe placement was manually adjusted to the sagittal plane of the muscle to obtain the clearest possible image in the plane parallel to at least three fascicles (Loram et al., 1985; Narici et al., 1996; Morse et al., 2005; Kanehisa et al., 2006; Klimstra et al., 2006; Benard et al., 2009; Konig et al., 2014; Bolsterlee et al., 2016). Scans were obtained at an ankle angle of ~90° at rest using a cast to passively maintain the position during scanning and to match the position in other participants. Intra-rater, between image and within-session reliability analysis, was excellent (May et al., 2021) and supports that this protocol can examine group differences for both the GM and GL.

Additional limitations of the study include the relatively small sample size, as high inter-individual variation can exist in muscle architecture. The observations of this study are only valid for gastrocnemius muscles. It may be more useful to explore adaptations in muscles that are more greatly activated, such as the quadriceps which is the primary muscle group for cycling (Ericson et al., 1985; Ryan and Gregor, 1992). As bi-articular muscles spanning two joints, the gastrocnemius medialis and lateralis length changes are dependent upon both the knee and ankle joints (Kawakami et al., 1998). Foot position and changing saddle height can result in increased ankle plantarflexion and knee extension (Ercison et al., 1988; Turpin and Watier, 2020). In this study, factors such as cyclist seat height, ankle or knee joint positions, and range, and participation in additional exercise requiring gastrocnemius activity such as walking, were not explored as covariates that are very likely to be highly variable between participants. The effects of these factors on resulting morphological adaptations cannot be separately identified in this cross-sectional study. Cross-sectional studies that compare populations with different training backgrounds are useful to evaluate the association between variables but are unable to determine cause and effect relationships. The genetic muscle morphology patterns of an athlete could have been suited to their respective sports from the outset, or muscles could have adapted over long-term training to become more suitable. Furthermore, the male and female groups of basketballers in this study exceeded the cyclists in terms of the level of competition, and training frequency per week. Unlike the males, many female basketballers had participated in other sports at a competitive level in their history, even though they had ceased well before this study. Whilst it remains possible that the results observed may reflect training history, it cannot be concluded that long-term sports-specific training systematically influences muscle morphology. Future research investigating the influence of sport-specific training on muscle architecture should compare athlete groups with similar training backgrounds, in addition to anthropometrics and age. Following the recent narrative review that concluded that the muscle length at which training is performed and intensity of training may be important factors that influence adaptations (Kruse et al., 2021), it is recommended that these be observed or directly controlled in future studies. Longitudinal studies might provide useful information in addition to cross-sectional comparisons, particularly if muscle architecture could be measured at baseline when the athlete first begins high-level training in their selected sport.

In conclusion, participation in selected competitive sports was associated with differences in gastrocnemius medialis and lateralis muscle architecture in female athletes; however, participation in concentric dominant muscle action, or a combination of concentric and eccentric muscle action, did not systematically influence gastrocnemius muscle architecture. Further research is required to improve the understanding of architectural adaptations in response to training muscle architecture in all regions of the skeletal muscle system.

## Data Availability Statement

The datasets presented in this study can be found in online repositories. The names of the repository/repositories and accession number(s) can be found below: Open At La Trobe; 2021. https://doi.org/10.26181/612f0b7259195.

## Ethics Statement

The studies involving human participants were reviewed and approved by La Trobe University Human Research Ethics Committee. The participants provided their written informed consent to participate in this study.

## Author Contributions

SM, SL, and MK contributed to the conceptualization and design of the study. MK contributed to resources and software, and alongside SL contributed to supervision. SM performed the investigations and data curation. Together, SM and MK contributed to formal analysis and validation. SM prepared the original draft manuscript. All authors contributed to manuscript revision, read, and approved the submitted version.

## Funding

This work was supported by an Australian Government Research Training Program Scholarship. The authors would also like to acknowledge the Bendigo Tertiary Education Anniversary Foundation and La Trobe University Holsworth Research Initiative's support of Professor Kingsley's research.

## Conflict of Interest

The authors declare that the research was conducted in the absence of any commercial or financial relationships that could be construed as a potential conflict of interest.

## Publisher's Note

All claims expressed in this article are solely those of the authors and do not necessarily represent those of their affiliated organizations, or those of the publisher, the editors and the reviewers. Any product that may be evaluated in this article, or claim that may be made by its manufacturer, is not guaranteed or endorsed by the publisher.
